# Midlife Growth Hormone Receptor Ablation Extends Healthy Lifespan and Induces Sex‐Specific Hepatic Transcriptional Changes at Single‐Cell Resolution

**DOI:** 10.1111/acel.70695

**Published:** 2026-09-05

**Authors:** Silvana Duran‐Ortiz, Edward O. List, Jonathan A. Young, Yuji Ikeno, Shouan Zhu, Todd McHugh, Patrick M. O'Connor, Minhoo Kim, Bérénice A. Benayoun, Fabian Benencia, Reetobrata Basu, Emmanuel A. Gotte, Darlene E. Berryman, John J. Kopchick

**Affiliations:** ^1^ Institute for Molecular Medicine and Aging, Heritage College of Osteopathic Medicine Ohio University Athens Ohio USA; ^2^ Ohio University Heritage College of Osteopathic Medicine, Ohio University Athens Ohio USA; ^3^ Department of Biomedical Sciences Ohio University Athens Ohio USA; ^4^ Diabetes Institute Ohio University Athens Ohio USA; ^5^ Sam and Ann Barshop Institute for Longevity and Aging Studies San Antonio Texas USA; ^6^ Department of Pathology and Laboratory Medicine The University of Texas Health Science Center at San Antonio San Antonio Texas USA; ^7^ Research Service, Audie L. Murphy VA Hospital South Texas Veterans Health Care System San Antonio Texas USA; ^8^ Ohio Musculoskeletal and Neurological Institute (OMNI) Ohio University Athens Ohio USA; ^9^ Ohio Center for Ecological and Evolutionary Studies Athens Ohio USA; ^10^ Leonard Davis School of Gerontology University of Southern California Los Angeles California USA; ^11^ USC Norris Comprehensive Cancer Center Los Angeles California USA; ^12^ USC Stem Cell Initiative Los Angeles California USA; ^13^ Molecular and Cellular Biology Program Ohio University Athens Ohio USA

**Keywords:** cre‐lox, growth hormone, healthspan, IGF‐1, lifespan, liver, snRNAseq

## Abstract

Suppression of growth hormone (GH) signaling is known to be effective to extend lifespan in mammals, yet most models rely on congenital disruption of the GH/insulin‐like growth factor‐1 (IGF‐1) axis. Whether modulation of this pathway later in life can still influence aging and the underlying cellular mechanisms remains incompletely understood. To address this, we ablated the growth hormone receptor (*Ghr*) at 12‐months of age in mice (12mGHRKO), using a tamoxifen‐inducible model. Midlife *Ghr* disruption produced the expected endocrine signature of GH resistance, including reduced circulating IGF‐1 and elevated GH levels. Importantly, lifespan was significantly extended in both sexes without major effects on somatic growth. Despite increased adiposity, male 12mGHRKO mice exhibited improved insulin sensitivity and protection against age‐related deterioration of neuromuscular performance and bone microarchitecture. Single‐nucleus RNA sequencing (snRNA‐seq) of liver tissue identified a reduction of B‐cells in both sexes and a dimorphic transcriptional remodeling, including a shift toward feminized gene expression in male hepatocytes, marked by reduced male‐biased gene expression and increased female‐biased transcriptional programs, consistent with impaired pulsatile GH‐STAT5 signaling. Together, these findings demonstrate that suppression of GH signaling initiated in middle age is sufficient to reshape hepatic transcriptional programs and promote healthy longevity, supporting the GH/IGF‐1 axis as a promising target for gerotherapeutic interventions.

## Graphical abstract

Reducing growth hormone receptor signaling beginning in middle age is sufficient to extend lifespan in both sexes while preserving metabolic and physical function, particularly in males. Single‐nucleus RNA sequencing reveals sex‐specific hepatic transcriptional remodeling associated with these longevity benefits.

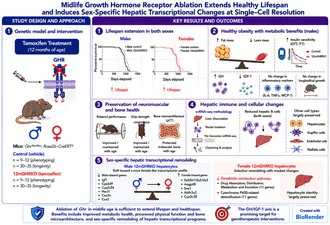

## 1 Introduction

Growth hormone (GH) plays a multifaceted role in physiology by stimulating somatic growth, promoting hepatic and peripheral insulin‐like growth factor‐1 (IGF‐1) production, and exerting both anabolic and catabolic effects in a tissue‐specific manner (Vijayakumar et al. 2010). While GH supports growth and metabolism, it also impairs insulin action, conferring a diabetogenic effect (Vijayakumar et al. 2010), and does have extra‐pituitary tissue‐specific expression, increasing with age and driving age‐associated co‐morbidities in an autocrine/paracrine manner (Chesnokova et al. 2025). Importantly, a conserved link between GH/IGF‐1 signaling and aging has emerged across species (Qian et al. 2022). In humans, similar associations have been observed; for example, certain Ashkenazi Jewish centenarians and their offspring possess IGF‐1 receptor mutations associated with reduced GH/IGF‐1 signaling (Suh et al. 2008). Likewise, individuals with Laron Syndrome (LS), who carry inactivating mutations in the GH receptor (GHR), display remarkable resistance to cancer, cognitive decline and diabetes, and exhibit reduced risk factors of cardiovascular disease and levels of aging biomarkers (Guevara‐Aguirre et al. 2024; Nashiro et al. 2017).

In mice, germline disruption of the GH/IGF‐1 axis as seen in Ames, Snell, lit/lit, and GH knockout (GH−/−) leads to health and lifespan benefits (Bartke and Darcy 2017). In fact, the most potent method for lifespan extension in laboratory mice to date is germline growth hormone receptor (GHR) gene disruption as the GHR knockout (KO) mouse is recognized as the world's longest‐lived laboratory mouse (Pilcher 2003). The GHRKO mice also exhibit enhanced healthspan, including improved strength, balance, motor coordination, cognitive function, protection from age‐associated osteoarthritis, improved insulin sensitivity, resistance to diabetes and cancer, and decreased markers of aging such as adipose tissue (AT) senescence and mTORC1 signaling in liver, kidney, heart and muscle (Fang et al. 2018; Liu et al. 2024; Qian et al. 2022; Stout et al. 2014). These findings, albeit from congenital models, underscore the effectiveness of targeting the GH/IGF‐1 axis as a therapeutic approach to promote healthy aging. However, for clinical relevance, anti‐aging interventions must be applied during adulthood to avoid interfering with early growth and development. To address this, our laboratory developed a series of adult‐onset tamoxifen‐inducible GHR knockout mice using the Cre‐lox system under the control of the ubiquitous ROSA26 promoter/enhancer (Duran‐Ortiz et al. 2021; Junnila et al. 2016). These mice have postnatal disruption of the *Ghr* gene at 1.5 and 6 months of age (equivalent to ~13 and ~30 years in humans, respectively) (Duran‐Ortiz et al. 2021; Dutta and Sengupta 2016; Junnila et al. 2016) and have an extended lifespan in females and improved markers of metabolic health in males, including reduced cancer incidence, improved insulin sensitivity, and tissue‐specific reductions in lipid and protein oxidation (Duran‐Ortiz et al. 2021) (Junnila et al. 2016).

Although congenital and early adult suppression of GH signaling robustly extends lifespan, it remains unclear how late in life GH action can be reduced while still producing beneficial effects. Defining this therapeutic window is particularly important because interventions targeting aging in humans would likely begin after completion of growth and development rather than during early life. Therefore, building on our previous findings, we evaluated the impact of *Ghr* gene disruption at 12 months of age, a widely accepted stage corresponding to middle age in mice (equivalent to ~40 years in humans) (Dutta and Sengupta 2016), to determine whether reducing GH action after normal growth and reproductive maturity remains sufficient to influence lifespan and healthspan (rotarod test, grip strength, frailty index and bone microarchitecture). Furthermore, to define the underlying molecular mechanisms at the cellular level, we performed single‐nucleus RNA sequencing (snRNA‐seq) on liver tissue from male and female 12mGHRKO and control mice. Together, this study not only tests the feasibility of delaying GHR‐targeted interventions until middle age but also provides a deeper insight into the sex‐specific hepatic transcriptional programs that may underlie the observed benefits in healthspan and longevity. By combining clinically relevant timing of intervention with an array of phenotypic assessments and advanced transcriptomic analysis, this work aims to advance the translational potential of GH‐targeted gerotherapeutics for human aging interventions.

## 2 Results

### 2.1 Midlife Reduction of GH Action Enhances Longevity in Both Sexes Without Affecting Growth

**FIGURE 1:**
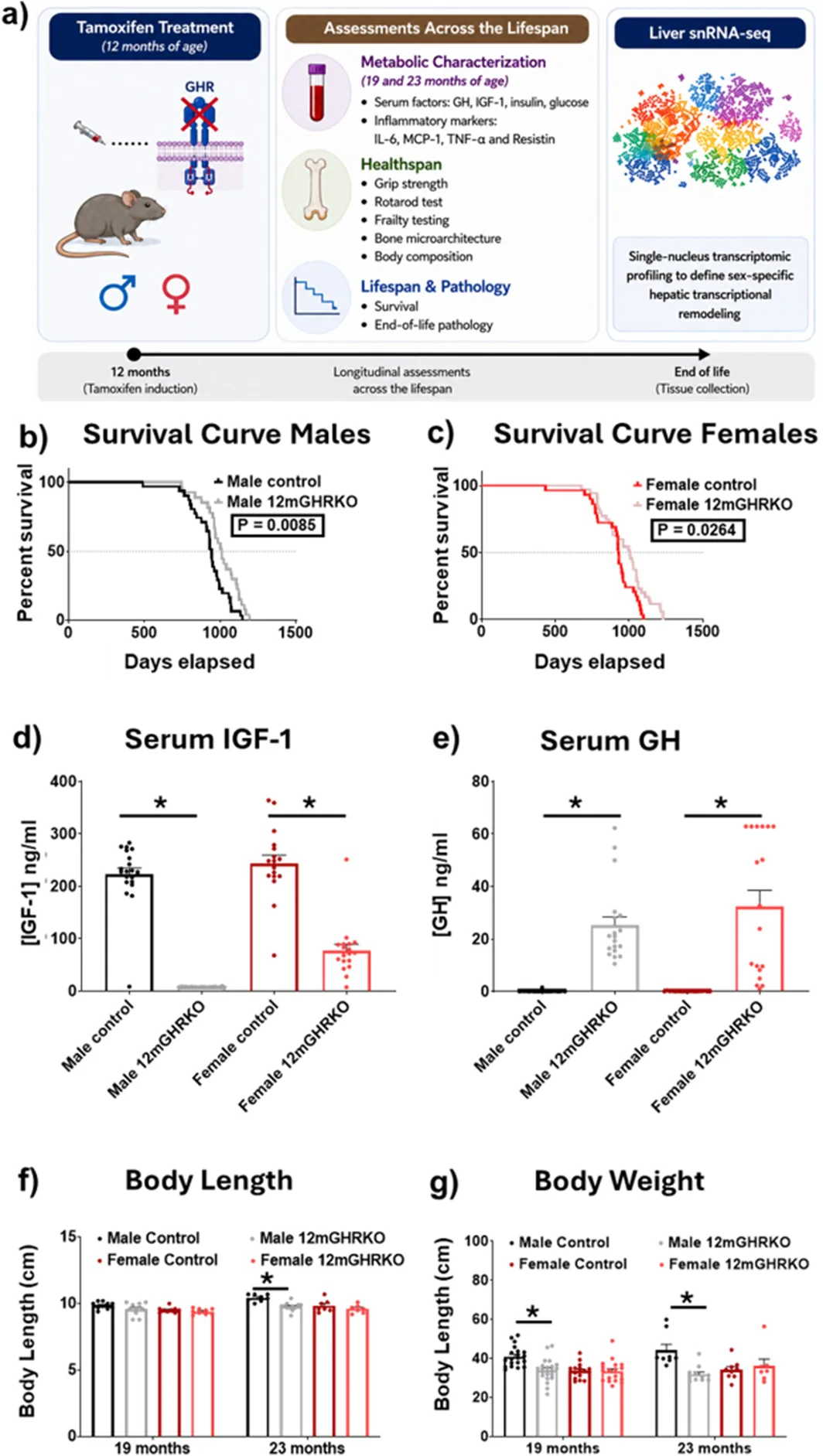
Midlife disruption of GHR signaling alters the GH/IGF‐1 axis and extends lifespan without impairing somatic growth. (a) Visual representation of the experimental design to evaluate the metabolic parameters, and the healthspan and lifespan of mice with the *Ghr* gene disrupted at 12 months of age (12mGHRKO mice). (b, c) Kaplan–Meier survival curves for male (a) and female (b) mice comparing control and 12mGHRKO animals. (d, e) Circulating IGF‐1 (d) and GH levels (e) measured in serum of male and female control and 12mGHRKO mice at 18 months of age. (f, g) Body length (f) and body weight (g) measurements of male and female control and 12mGHRKO mice at the indicated ages. Panel (a) was generated with Biorender. Gray symbols/bars represent control mice and red symbols/bars represent 12mGHRKO mice. Individual points represent individual animals. All values are shown as mean ± SE. Student two‐tailed *t*‐tests were used to assess differences between 12mGHRKO and control mice within each sex. Survival curves were analyzed using the log‐rank test.

To reduce GH action in middle‐aged mice, we used the Cre‐Lox system driven by the Rosa26 locus to ablate the *Ghr* gene in male and female mice of 12 months of age, referred to as 12mGHRKO mice. Longevity studies and end‐of‐life pathology were performed in these mice (Figure 1a), and a log‐rank test showed significant lifespan extension in male (*p* = 0.0085) and female (*p* = 0.0264) 12mGHRKO compared to control mice (Figure 1b,c). The 12mGHRKO females showed an 8% increase in median survival, with 1003 days compared to 929 days in female controls (*p* = 0.0492; Tables S1 and S2). Maximal lifespan in female 12mGHRKO mice was extended by 12%, with the longest‐lived female 12mGHRKO surviving 1231 days compared to 1097 days for controls (*p* = 0.0280; Table S1). To provide a more robust assessment of maximal lifespan, we additionally performed the maximal lifespan analysis described by Wang et al. (2004). Consistent with our Kaplan–Meier percentile survival analyses, female 12mGHRKO mice exhibited a significantly greater proportion of animals surviving into the longest‐lived 10% of the pooled population compared with female controls (*p* = 0.0133; Table S6). Although not significant, median survival for male 12mGHRKO was extended 7% with median lifespan being 1011 days for the 12mGHRKO male mice and 943 days for male controls (*p* = 0.0643; Tables S1 and S2). Maximal lifespan in 12mGHRKO and control males was not significantly different at 1194 and 1147 days, respectively (*p* = 0.1734, Tables S1 and S2). The Wang and Allison maximal lifespan analysis likewise showed no significant difference between male 12mGHRKO and control mice (*p* = 0.4023; Table S5), indicating that the longevity benefit in males was primarily reflected by an extension of median rather than maximal lifespan. Interestingly, despite the longer lifespan, end‐of‐life pathology analysis did not show significant changes in the incidence of fatal neoplastic lesions or disease burden of 12mGHRKO mice in either sex compared to controls (Table S3).

To perform the metabolic characterization and healthspan assessment of the 12mGHRKO and control mice, we had two separate experimental groups that were monitored and sacrificed at 19‐ or 23‐ months of age (7‐ or 11‐months post *Ghr* gene ablation—Figure 1a). To test the efficacy of GHR ablation in 12mGHRKO mice, we evaluated the levels of circulating IGF‐1, a primary readout of GH signaling, and GH at 19 months of age (Figure 1d,e). Male and female 12mGHRKO mice were GH insensitive, showing significant reduction in circulating IGF‐1 (greater reduction in males vs. females), along with elevated GH levels when compared to control mice (*p* < 0.0001; Figure 1d,e). We further evaluated *Ghr* and *Igf‐1* mRNA expression in multiple tissues at 19‐ and 23‐ months of age (Figure S1). *Ghr* gene expression was significantly reduced at both ages in all tissues in both sexes, with the liver and subcutaneous AT (subq) being the most susceptible to *Ghr* ablation (*p* < 0.0001; Figure S1). Interestingly, *Igf‐1* gene expression followed the trend of *Ghr* gene expression, in liver and subq, but not in kidney and quad tissues (Figure S1).

GH is required for longitudinal growth and development, and either excess or suppression in GH action leads to supra‐normal or sub‐normal stature, respectively. Therefore, we evaluated body length and body weight at 19‐ and 23‐months of age; ablation of GHR in middle age had no impact on body length at 19 months of age but a 6% reduction was observed at 23 months of age in 12mGHRKO males compared to controls (Figure 1f, *p* < 0.0001). Interestingly, body weight was unchanged in 12mGHRKO females but reduced in 12mGHRKO males compared to controls at both time points (Figure 1g, *p* < 0.0001). Repeated measures ANOVA of body weight over time confirmed this, showing significant changes in body weight in male 12mGHRKO mice starting at 4‐weeks post *Ghr* gene disruption (Figure S2a,e).

### 2.2 Inhibition of GH Action in Middle Aged Males Improves Insulin Sensitivity Despite Obesity

**FIGURE 2:**
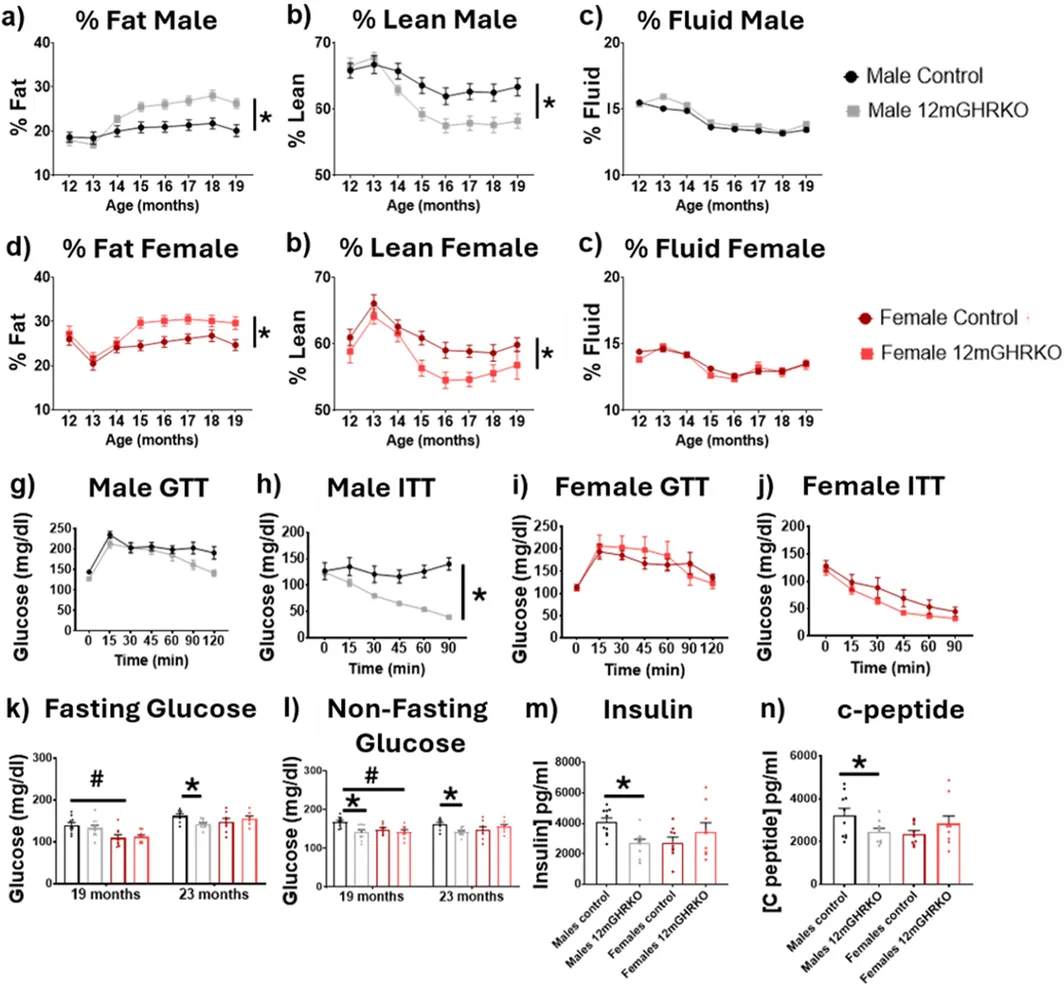
Midlife disruption of GH signaling alters body composition and improves insulin sensitivity. (a–f) Longitudinal body composition analysis in male and female 12mGHRKO and control mice showing percentage fat mass (a, d), percentage lean mass (b, e), and percentage fluid (c, f) over time. (g, h) Glucose tolerance tests (GTT), and insulin tolerance tests (ITT) performed in male (g, h) and female (i, j) 12mGHRKO and control mice at 23 months of age. (k–n) Measurements of metabolic parameters including fasting glucose (k), non‐fasting glucose (l), and serum insulin (m), and serum c‐peptide (n) levels in male and female 12mGHRKO and control mice at 19‐months and 23‐months of age. Gray symbols/bars represent control mice and red symbols/bars represent 12mGHRKO mice. Individual points represent individual animals. All values are mean ± SE. Repeated‐measures two‐way ANOVA was used for longitudinal body composition and glucose tolerance/insulin tolerance tests. Student two‐tailed *t*‐tests were used for comparisons between groups within each sex. *p ≤ 0.05.

GH action changes body composition as it promotes AT lipolysis (catabolic effect) while promoting glucose production, protein synthesis and muscle growth (anabolic effect) (Vijayakumar et al. 2010). Because of this we assessed body composition over time in male and female mice and their respective controls (Figure 2a–f, Figure S2). 12mGHRKO mice showed increased fat mass percentage (Figure 2a,d, *p* < 0.0001) and reduced lean mass percentage (Figure 2b,e, *p* < 0.0004) compared to control mice, while percentage of fluid was not altered in 12mGHRKO mice (Figure 2c,f). However, when not normalized to body weight, only male lean mass was significantly changed between 12mGHRKO mice and controls (Figure S2c). Because of the increased fat mass seen in 12mGHRKO mice, we assessed serum inflammatory markers in these mice at 19 months of age (Figure S3). Only resistin showed a significant increase in 12mGHRKO males compared to controls (*p* = 0.0133), while Interleukin‐6 (IL‐6), Monocyte Chemoattractant Protein‐1 (MCP‐1) and Tumor Necrosis Factor alpha (TNF‐α) were unaffected between 12mGHRKO mice and controls (Figure S3).

Because of the changes in body composition and to test whether decreased GH action in middle‐aged mice also influences organ development, we measured tissue weights at 19 and 23 months of age (Figure S4). We found that when normalized to body weight, only the spleen of male 12mGHRKO mice at 23 months of age was significantly reduced compared to controls (Figure S4a). However, a sex‐ and age‐dependent reduction in the total weight of all internal organs (pancreas, liver, kidney, lung, and intestine) and skeletal muscles (gastrocnemius and soleus) (*p* ≤ 0.0500) was found in 12mGHRKO mice (Figure S4b). At 19 months of age, total subq AT weight was significantly higher in male and female 12mGHRKO mice compared to controls (*p* ≤ 0.05). Interestingly, at 23 months of age, except for quadriceps, male 12mGHRKO had reduced weights (non‐normalized) of all internal organs and skeletal muscles compared to controls (Figure S4b).

Due to GH's diabetogenic effect and impact on glucose homeostasis, we performed glucose tolerance tests (GTTs) and insulin tolerance tests (ITTs) at 19‐ and 23‐months of age (Figure 2f–j, Figure S5). Insulin sensitivity was significantly improved only in male 12mGHRKO mice compared to controls at both 19‐ and 23‐months of age (*p* < 0.0001, Figure 2h–j and Figure S4). Glucose tolerance was similar in both sexes at both time points (Figure 2g–i and Figure S4). Fasting glucose levels were reduced in female controls compared to male controls at 19 months of age whereas both fasting and non‐fasting glucose were significantly decreased in 12mGHRKO males compared to controls (*p* < 0.0100, Figure 2i–k). Congruent with this, at 19‐months of age, fasted serum insulin and c‐peptide were significantly lower in 12mGHRKO mice compared to controls (*p* < 0.0100, Figure 2m,n).

### 2.3 Attenuation of GH Action in Middle‐Aged Male Mice Protects Against Age‐Related Decline in Strength and Endurance

**FIGURE 3:**
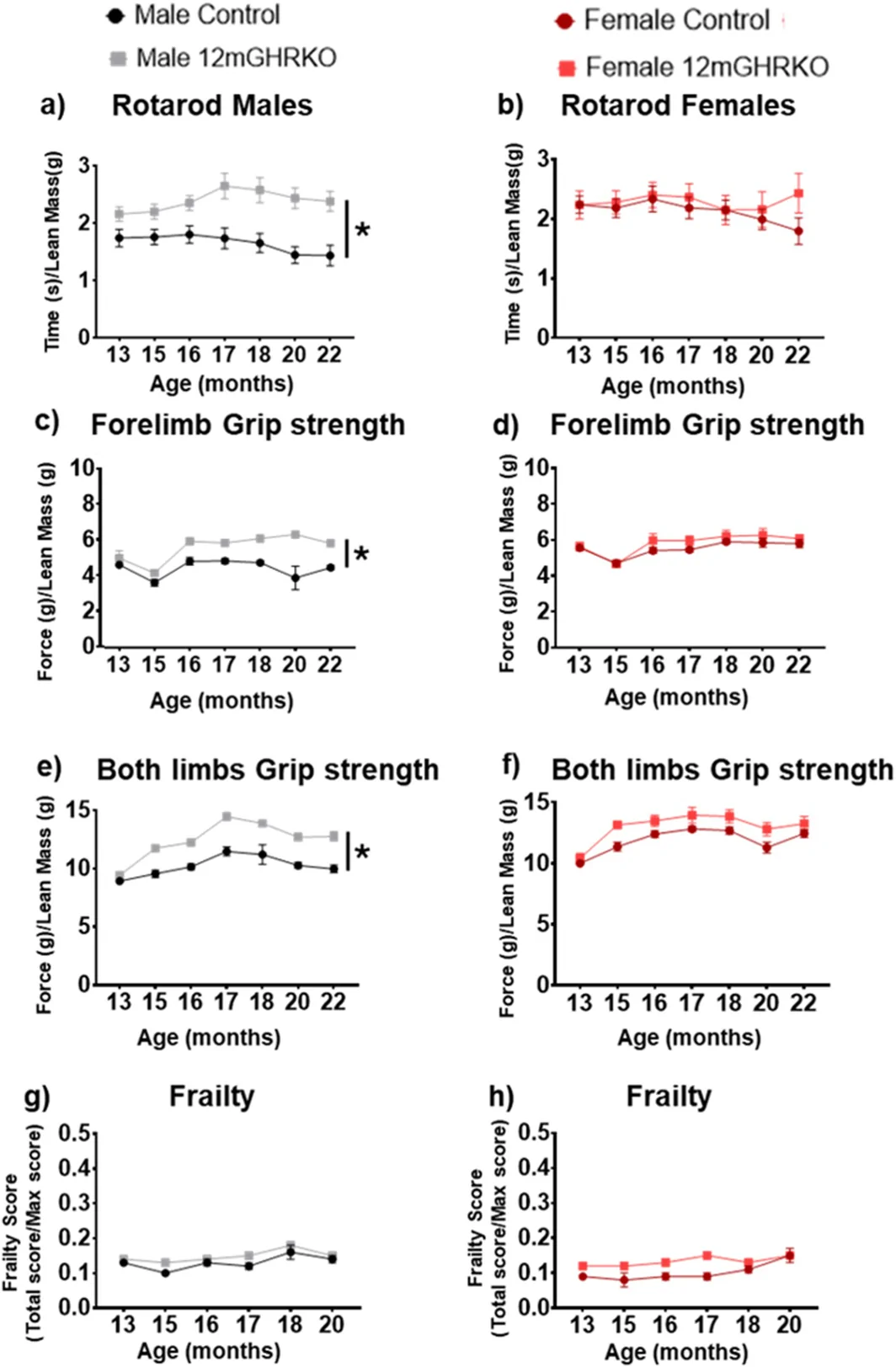
Midlife disruption of GH action preserves neuromuscular during aging. (a, b) Rotarod performance in male (a) and female (b) mice over time. (c, d) Forelimb grip strength normalized to lean mass in male (c) and female (d) mice over time. (e, f) Hindlimb grip strength normalized to lean mass in male (e) and female (f) mice over time. (g, h) Frailty index scores in male (g) and female (h) mice assessed longitudinally with age. Gray symbols/bars represent control mice and red symbols/bars represent 12mGHRKO mice. Individual points represent individual animals. All values are mean ± SE. Repeated measures two‐way ANOVA was used to evaluate differences between groups over time. *p ≤ 0.05.

To determine whether reduced GH action beginning at 12 months of age influences functional health during aging, we assessed neuromuscular performance and frailty longitudinally in male and female 12mGHRKO mice and their respective controls (Figure 3 and Figure S6). Motor coordination and balance were evaluated using the rotarod test, while muscle strength was assessed using forelimb and hindlimb grip strength tests. Because 12mGHRKO mice exhibit reduced lean mass compared to controls (Figure 2a–f), rotarod performance and grip strength measurements were normalized to lean mass to account for differences in muscle mass between groups (Figure 3a–f).

While rotarod performance and grip strength were improved in 12mGHRKO males, these measurements were not altered in female 12mGHRKO mice compared to controls throughout aging (*p* < 0.0500, Figure 3a–f). Frailty was evaluated using a non‐invasive validated mouse frailty index (Parks et al. 2012), which quantifies the accumulation of multiple age‐associated health deficits and serves as an integrated measure of biological aging and healthspan. Frailty scores showed no significant difference between 12mGHRKO mice and control mice throughout the measurement period (Figure 3g,h).

### 2.4 Midlife GHR Disruption Preserves Vertebral Trabecular Bone Microarchitecture in Males

**FIGURE 4:**
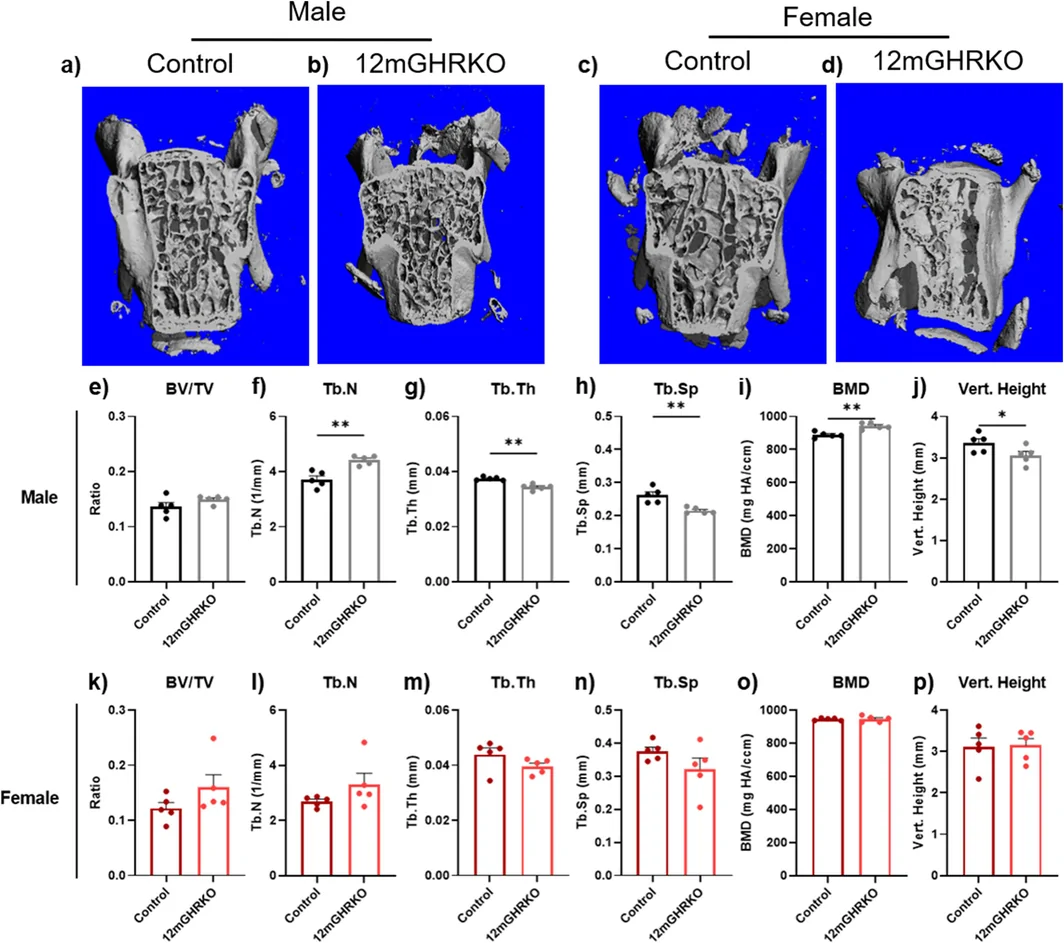
Midlife disruption of GH signaling modestly preserves vertebral trabecular bone microarchitecture. (a‐d) Representative three‐dimensional micro–computed tomography (μCT) reconstructions of vertebral trabecular bone from control and 12mGHRKO mice in males and females. (e–p) Quantitative μCT analysis of vertebral trabecular bone parameters of male (e–j) and female mice (k–p), including bone volume fraction (BV/TV), trabecular number (Tb.N), trabecular thickness (Tb.Th), trabecular separation (Tb.Sp) and bone mineral density (BMD). Gray bars represent control mice and red bars represent 12mGHRKO mice. Individual points represent individual animals. All values are shown as mean ± SE. Student two‐tailed t tests were used to assess differences between 12mGHRKO and control mice within each sex. *p ≤ 0.05.

Because of the improved healthspan results in males and due to GH's role in skeletal growth and bone remodeling, we next evaluated whether disruption of GH action at 12 months of age influences bone structure. Vertebral trabecular bone architecture was assessed using micro‐computed tomography (μCT) in male and female 12mGHRKO mice and their respective controls at 19 months of age (Figure 4). Representative three‐dimensional reconstructions of the vertebral trabecular compartment are shown in Figure 4a–d. μCT analyses revealed sex‐dependent effects of midlife GHR disruption on trabecular bone parameters, with males being more affected than females. In males, 12mGHRKO mice had higher trabeculae number (Tb.N), lower trabecular bone thickness (Tb.Th), lower trabecular space (Tb.Sp), higher bone mineral density (BMD), and lower vertebral height (*p* < 0.0500, Figure 4e–j). These results suggest preservation of trabecular architecture. In females, reduced GH action in middle age did not impair vertebral bone microarchitecture as the μCT measurements were largely comparable between 12mGHRKO and control mice (Figure 4k–p). Although a slight trend toward reduced trabecular separation (Tb.Sp) was observed, this finding was not statistically significant (Figure 4n). BV/TV did not differ between 12mGHRKO and the control group for either sex.

### 2.5 Midlife GHR Disruption Remodels Hepatocyte Cellular States and Suppresses Canonical GH‐Responsive Programs

**FIGURE 5:**
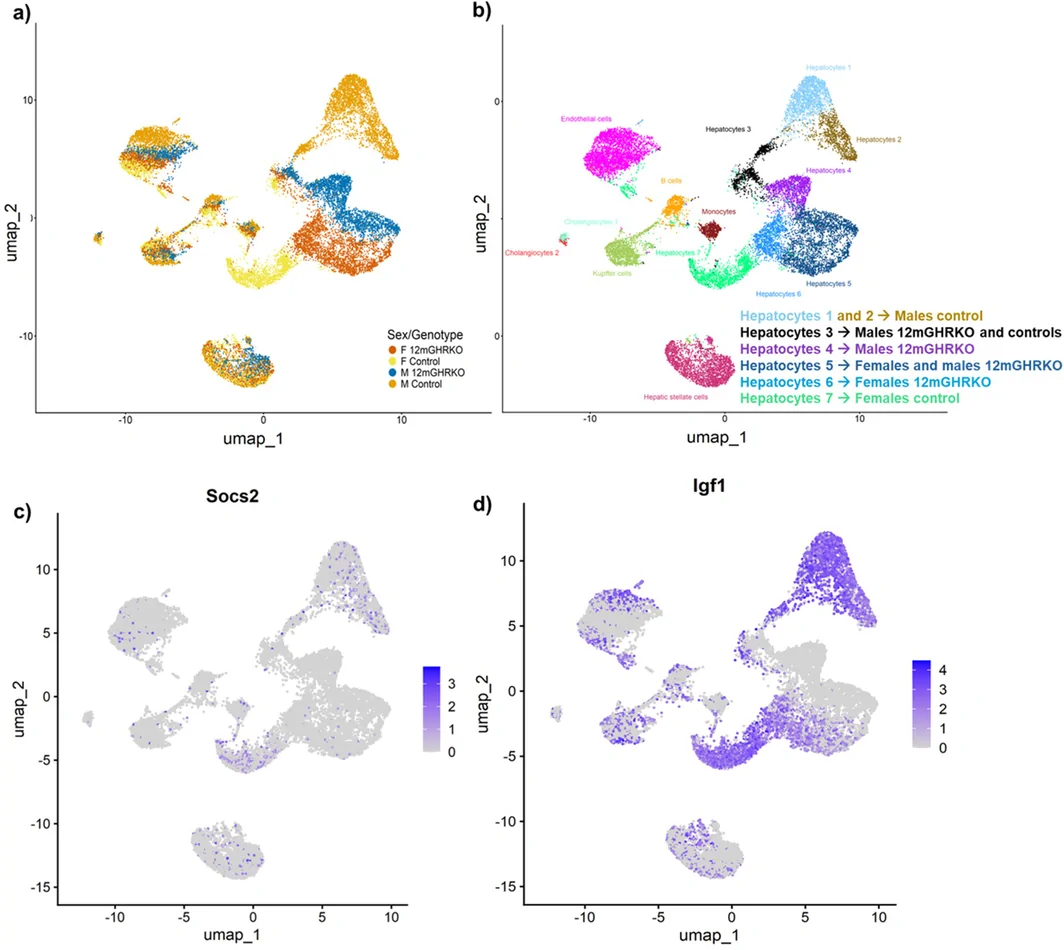
Midlife *Ghr* disruption induces sex‐specific hepatocyte clusters and reduces expression of canonical GH‐responsive genes in the liver. (a) Uniform Manifold Approximation and Projection (UMAP) of integrated single‐nucleus RNA sequencing (snRNAseq) data from livers of 19‐month‐old male and female control and 12mGHRKO mice. Each point represents a single nucleus and is colored by sex and genotype. (b) UMAP of the same dataset annotated by cell type, identifying major hepatic and non‐parenchymal populations, including seven hepatocyte subclusters, endothelial cells, Kupffer cells, cholangiocytes, B‐cells, monocytes, hepatic stellate cells, and oligodendrocytes. Hepatocyte subclusters displayed strong sex‐ and genotype‐specific enrichment, with hepatocyte clusters 1 and 2 predominantly composed of male control nuclei, cluster 3 enriched for male 12mGHRKO and male control nuclei, cluster 4 enriched for male 12mGHRKO nuclei, cluster 5 enriched for females and males 12mGHRKO and clusters 6–7 enriched for female 12mGHRKO and control nuclei, respectively. (c) Feature plot showing expression of Socs2 (Suppressor of Cytokine Signaling 2), a canonical GH‐STAT5 target gene, which was highly expressed in hepatocyte clusters associated with intact GH signaling and reduced in clusters enriched for 12mGHRKO nuclei. (d) Feature plot showing expression of Igf1 (Insulin Growth Factor‐1), the principal hepatic endocrine output of GH signaling, with lower expression in hepatocyte clusters enriched for 12mGHRKO nuclei.

To investigate tissue‐level mechanisms underlying the beneficial effects of adult‐onset GH resistance, we performed single‐nucleus RNA sequencing (snRNA‐seq) on liver samples collected from 12mGHRKO and control mice at 19 months of age. The liver was prioritized because it is a central effector organ of the GH/IGF‐1 axis, with one of the highest tissue‐level expressions of the GHR which induces the production of approximately 75%–90% of circulating IGF‐1 found in circulation (List et al. 2014). Cluster analysis identified the gene expression between sex and genotype (Figure 5a). Integrated clustering, which combined all samples into a shared analysis, identified the expected major hepatic cell populations, including hepatocytes, endothelial cells, Kupffer cells, hepatic stellate cells, cholangiocytes, B‐cells, and monocytes, confirming high‐quality capture of both parenchymal and non‐parenchymal compartments (Figure 5b). Hepatocytes were the predominant cellular population (53.9% of total nuclei), followed by endothelial cells (17.0%), hepatic stellate cells (13.5%), and immune cells (14.4% combined) (Table S4). Interestingly, the proportion of B‐cells was reduced in both male and female 12mGHRKO mice to approximately half that observed in controls. Male and female 12mGHRKO mice exhibited B‐cell frequencies of 3.8% and 1.4%, respectively, compared with 6.2% in male controls and 3.7% in female controls (Table S4).

Hepatocytes showed a large variation in gene expression profiles and were thus categorized into seven Hepatocyte Clusters (HC‐1 to HC‐7), while most other cell types were contained within a single cluster. Further examination of the various hepatocyte clusters identified several distinct hepatocyte states strongly associated with sex and genotype (Figure 5b). Clusters were enriched for female control (HC‐7), male control (HC‐11 and ‐2), female 12mGHRKO (HC‐6), and male 12mGHRKO hepatocytes (HC‐4), as well as intermediate clusters shared between groups for male and female 12mGHRKO mice (HC‐5) as well as male 12mGHRKO and control mice (HC‐3). Two male control enriched clusters were identified (HC‐1 and ‐2), suggesting multiple male‐specific transcriptional states under normal pulsatile GH signaling. These clusters represent hepatocytes with similar transcriptional programs rather than fixed cell types. Male control hepatocytes were concentrated almost exclusively within two control‐associated clusters (HC‐1 showed 29.3% and Hepatocytes cluster 2 had 17.3% of male control nuclei, respectively; Table S4). In contrast, 12mGHRKO mice showed emergence of genotype‐associated clusters, including female 12mGHRKO ‐dominant (HC‐6), male 12mGHRKO ‐dominant (HC4), and shared female 12mGHRKO/male 12mGHRKO hepatocyte populations (HC‐5), indicating that loss of GHR signaling generates new gene expression states rather than simply redistributing cells among existing control clusters.

Comparison of genotype distributions across hepatocyte clusters revealed marked sex differences in transcriptional plasticity following *Ghr* gene disruption (Figure 5b). Male 12mGHRKO hepatocytes showed the greatest redistribution, with reduced occupancy of male control specific clusters (HC‐1 and ‐2) and expansion of knockout‐associated states, including male 12mGHRKO hepatocytes (HC‐4, 26.8%) and the shared female 12mGHRKO/male 12mGHRKO cluster (HC‐5, 27.4%) (Figure 5b; Table S4). Female 12mGHRKO hepatocytes also shifted, but to a lesser extent, retaining greater representation in female‐associated states, including female 12mGHRKO hepatocytes (HC‐6, 18.1%) and the shared female 12mGHRKO/male 12mGHRKO cluster (HC‐5, 29.4%). These findings suggest that male hepatic transcriptional architecture is more dependent on intact GH signaling, consistent with the sexually dimorphic pulsatile GH secretion pattern in male rodents.

Effective suppression of hepatic GH signaling was confirmed with feature plots showing expression levels of GH target genes. Expression of Suppressor of Cytokine Signaling 2 *(Socs2)*, a canonical GH‐JAK2‐STAT5 target, was highest in wild‐type hepatocyte clusters and markedly reduced in knockout‐associated states (Figure 5c). Likewise, *Igf1*, the principal endocrine output of hepatic GH action, was robustly expressed in wild‐type hepatocytes but substantially reduced in knockout clusters (Figure 5d), consistent with reduced systemic GH/IGF‐1 axis signaling. Residual expression of *Igf1* in some knockout cells likely reflects incomplete recombination or transitional states. Together, these data show that midlife GHR disruption substantially remodels hepatocyte gene expression programs while suppressing canonical GH‐responsive signaling pathways.

### 2.6 Midlife GHR Disruption Induces Sex‐Specific Hepatic Transcriptional Remodeling and Partial Feminization of Male Hepatocytes

**FIGURE 6:**
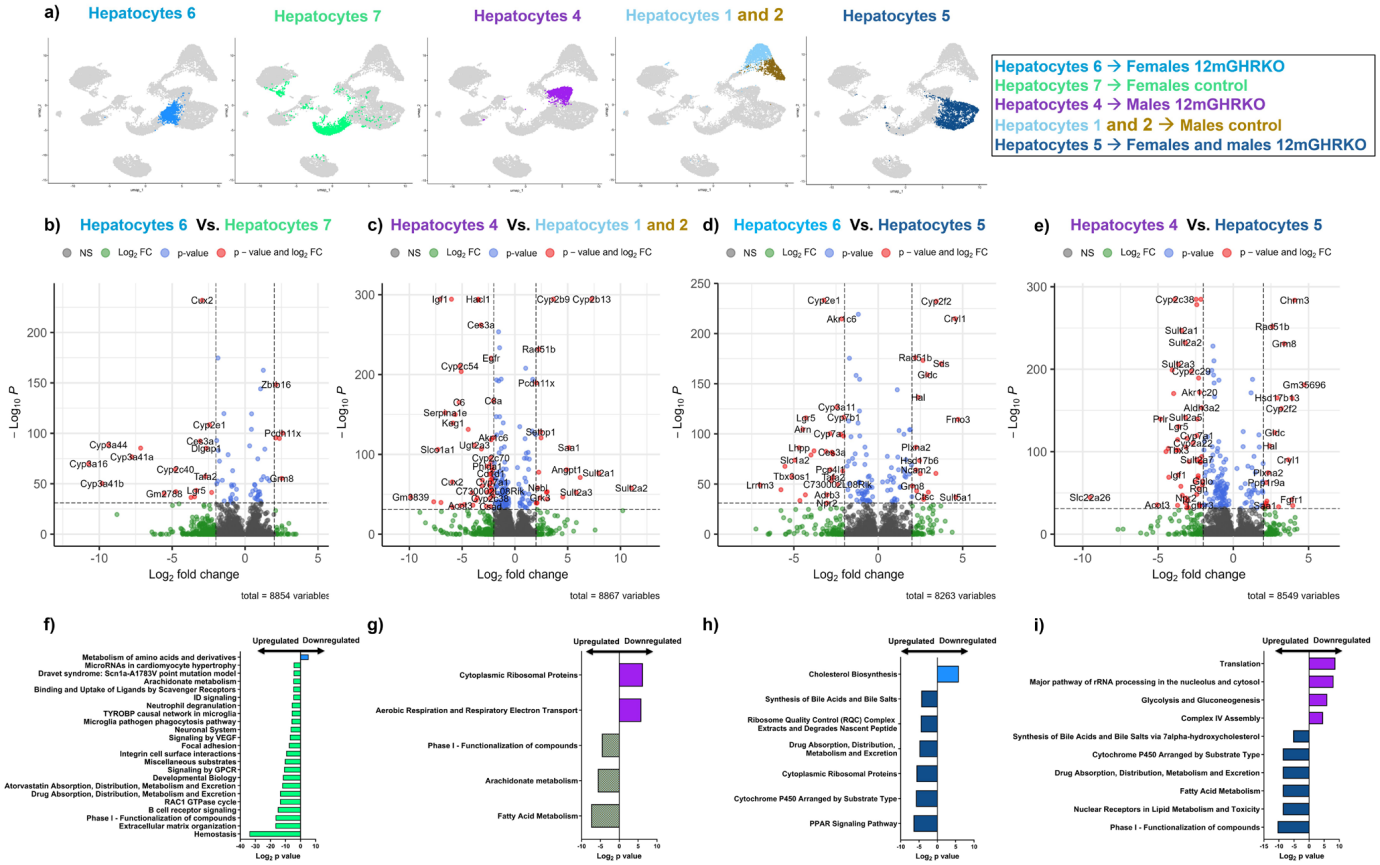
Sex‐Specific Hepatocyte Transcriptional Responses to Midlife *Ghr* Disruption Revealed by Single‐Nucleus RNA Sequencing (snRNAseq). snRNAseq analysis of hepatocyte subclusters from livers of control and 12mGHRKO mice demonstrating sex‐specific transcriptional remodeling following middle‐aged disruption of GHR signaling. Hepatocyte clusters were identified by gene expression profile. Colors correspond to dominant cluster compared: Hepatocytes 6, female 12mGHRKO; Hepatocytes 4, male 12mGHRKO; Hepatocytes 7, female control; Hepatocytes 5, mixed female and male 12mGHRKO; Hepatocytes 1 and 2, male control. (a) UMAP visualization of hepatocyte gene expression clusters enriched by sex and genotype. (b, f) Volcano plot and pathway analysis comparing female 12mGHRKO hepatocytes (cluster 6) with female control hepatocytes (cluster 7). (c, g) Volcano plot and pathway analysis comparing male 12mGHRKO hepatocytes (cluster 4) with male control hepatocytes (clusters 1 and 2). (d, h) Volcano plot and pathway analysis comparing female 12mGHRKO hepatocytes (cluster 6) with shared knockout‐associated hepatocytes (cluster 5). (e, i) Volcano plot and pathway analysis comparing male 12mGHRKO hepatocytes (cluster 4) with shared knockout‐associated hepatocytes (cluster 5). Differential expression analyses were performed between indicated hepatocyte clusters. Volcano plots display log2 fold change versus –log10 adjusted *p* value. Pathway enrichment analyses show representative significantly altered biological pathways for each comparison.

To define transcriptional changes associated with loss of GH signaling at middle age, we performed differential expression and pathway analyses across genotype‐ and sex‐specific hepatocyte clusters identified by snRNA‐seq (Figure 6a). Female 12mGHRKO hepatocytes (HC‐6) exhibited a comparatively modest change in gene expression relative to female controls (HC‐7) (Figure 6b), with reduced expression of several cytochrome P450 genes involved in steroid and xenobiotic metabolism, including Cyp3a16, Cyp3a41a, Cyp3a44, and Cyp2e1, alongside increased expression of Zbtb16, Pcdh11x, Grm8, and Cux2 (Figure 6b). Pathway enrichment analysis identified down‐regulation of xenobiotic metabolism pathways, including Drug Absorption, Distribution, Metabolism and Excretion and cytochrome P450‐related detoxification programs involving 11 genes (Figure 6f; Table S5), suggesting selective metabolic remodeling in females without widespread disruption of hepatocyte identity.

In contrast, male 12mGHRKO hepatocytes (HC‐4) displayed substantially broader transcriptional reprogramming when compared with male control hepatocytes (HC‐1 and HC‐2) (Figure 6c). Multiple female‐biased sulfotransferase genes, including Sult2a1, Sult2a2, and Sult2a3, were strongly induced, together with Angptl8 and Saa1, genes linked to lipid metabolism and signaling. At the same time, several canonical male‐enriched and GH‐responsive genes were markedly suppressed, including Igf1, the principal endocrine output of hepatic GH signaling, as well as Cyp2d9, Cyp2c54, Hacl1, Ces3a, and Cux2. Additional changes included altered expression of genes involved in DNA repair and cellular stress responses, such as Rad51b, Setbp1, and Ugt2a3. Pathway analysis revealed down‐regulation of lipid metabolism, xenobiotic processing, and endocrine signaling pathways, including Phase I Functionalization of Compounds, which contained 24 genes associated with xenobiotic metabolism (Figure 6g; Table S5).

Comparisons between knockout‐enriched hepatocyte states further supported convergence of male 12mGHRKO hepatocytes toward a more female‐like phenotype. Relative to the shared knockout‐associated hepatocyte cluster (HC‐5), male 12mGHRKO hepatocytes retained persistent enrichment of female‐associated genes (Figure 6e), including Sult2a family members, Aldh3a2, and Cyp2c38, while also showing differential expression of Chrm3, Gm35696, Hsd17b13, and Fgfr1. Similarly, comparison of female 12mGHRKO hepatocytes (HC‐6) with the shared knockout‐associated hepatocyte cluster (HC‐5) demonstrated continued suppression of several male‐biased metabolic genes (Figure 6d), with pathway analysis showing down‐regulation of Drug Absorption, Distribution, Metabolism and Excretion and Cytochrome P450 Arranged by Substrate Type involving 11 genes, supporting continued sex‐dependent specialization of knockout‐associated hepatocyte states (Figure 6h–i; Table S5). Collectively, these findings demonstrate that adult‐onset disruption of GH signaling in middle age produces greater hepatic transcriptional remodeling in males than females and induces partial feminization of sexually dimorphic liver gene expression programs.

## 3 Discussion

While lifelong reduction of GH signaling is well established to extend lifespan across multiple species (Bartke and Darcy 2017), the extent to which modulation of this pathway later in life remains beneficial has been less clear despite being of significant clinical relevance and interest. The present study demonstrates that disruption of GHR signaling beginning in *middle age* extends lifespan in both male and female mice and improves multiple aspects of metabolic (e.g., organ size, insulin sensitivity and bone micro‐architecture) and functional (e.g., rotarod and grip strength tests) health without substantially altering longitudinal growth. Importantly, the intervention was initiated at 12 months of age, corresponding approximately to middle age in humans, providing a clinically relevant time window to evaluate the translational potential of targeting the GH/IGF‐1 axis as a gerotherapeutic strategy. Notably, liver snRNAseq revealed a shift in B‐cell proportions and pronounced transcriptional changes in hepatocytes following midlife GHR disruption, including alterations in xenobiotic metabolism pathways and a partial feminization of the male hepatic gene expression program. These findings suggest that systemic longevity benefits observed after midlife suppression of GH signaling may arise, in part, from endocrine‐driven reprogramming of metabolic tissues. Although 12 months represents middle age rather than advanced age in mice, this intervention occurs after completion of somatic growth and reproductive maturity, allowing us to isolate the contribution of GH signaling during aging independent of developmental effects. Future studies examining *Ghr* disruption at older ages will be important to define the latest effective intervention window and further enhance clinical relevance.

Consistent with effective disruption of GHR signaling, 12mGHRKO mice displayed the endocrine signature of GH resistance, including reduced circulating IGF‐1 levels accompanied by compensatory elevations in serum GH (Laron et al. 1966). This reflects loss of hepatic IGF‐1 feedback on GH secretion and is a hallmark of impaired hepatic GHR signaling (Laron et al. 1966). Importantly, despite a state of reduced GH action, somatic growth was largely preserved with only modest changes in body length late in life, presumably because mouse growth plates never completely fuse (Jilka 2013). This contrasts sharply with germline GHR knockout models and with 1.5mGHRKO mice, which display reduced body size due to disruption of GH signaling during development (Junnila et al. 2016; Qian et al. 2022). These findings demonstrate that aging remains responsive to GH manipulation even after a major somatic growth period has ceased.

One of the most notable findings is the extension of lifespan following midlife disruption of GH action. Both sexes exhibited an increase in lifespan, consistent with evidence that reduced GH/IGF‐1 signaling is a robust longevity intervention (Bartke and Darcy 2017). Germline GHRKO mice remain the longest‐lived laboratory mouse model (with one mouse living ~5 years of age) (Pilcher 2003), and other GH‐deficient or resistant models, including Ames dwarf (Brown‐Borg et al. 1996), Snell dwarf (Flurkey et al. 2002), GHRH‐KO (Sun et al. 2013), GH‐deficient mice (Lasher et al. 2024), and GHR antagonist models, also display remarkable lifespan extension. However, the magnitude of lifespan extension observed here is more modest than that reported in congenital GH‐deficient or resistant models (20%–60% extension) (Qian et al. 2022), likely reflecting timing of intervention. Germline disruption of GH signaling influences developmental and lifelong physiological processes that cumulatively contribute to the exceptional longevity observed in those models (Bartke and Darcy 2017). Adult interventions occur after key physiological trajectories are established, thereby limiting aging biology remodeling. Nevertheless, the fact that suppression of GH action beginning as late as middle age extends lifespan underscores the GH/IGF‐1 axis as a promising target for clinically relevant gerotherapeutic interventions.

Previous work from our laboratory demonstrated that adult‐onset disruption of *Ghr* earlier in adulthood (1.5‐ or 6‐months of age) extends lifespan primarily in females while improving metabolic parameters in males (Duran‐Ortiz et al. 2021; Junnila et al. 2016). Notably, males in the 6mGHRKO mice did trend toward increased lifespan, suggesting the possibility of limited statistical power rather than absence of effect (Duran‐Ortiz et al. 2021). This interpretation is consistent with studies indicating that larger cohort sizes are often required to detect longevity effects in males (Zhavoronkov et al. 2025). Additional evidence shows that late‐life reduction of IGF‐1 signaling extends lifespan in female mice (Ashpole et al. 2017; Bale et al. 2017), while adult‐onset isolated GH deficiency improves insulin sensitivity (Cordoba‐Chacon et al. 2014). Together, these studies indicate that adult modulation of this axis remains sufficient to influence aging, albeit in a sex‐dependent manner. In contrast to germline GHRKO mice and earlier adult‐onset models, we did not observe a reduced neoplasm incidence in the 12mGHRKO cohort. This may reflect the timing of intervention, as oncogenesis may be initiated prior to intervention (Trastus and d'Adda di Fagagna 2025), limiting the ability of subsequent suppression of GH action to overtly modify oncogenic trajectories. However, despite a longer survival, malignancy burden was not increased in 12mGHRKO mice, suggesting a generalized slowing of aging rather than disease‐specific protection. Thus, 12mGHRKO mice living longer but succumbing to similar pathologies is consistent with delayed biological aging.

In line with these observations, sex‐specific longevity responses to GH modulation are likely associated with fundamental differences in endocrine physiology between males and females. In rodents, males display pulsatile GH secretion whereas females show continuous secretion (Jansson et al. 1985), resulting in sexually dimorphic STAT5‐dependent transcriptional programs in the liver (Zhang et al. 2012). Furthermore, estrogen suppresses GH signaling via induction of SOCS‐2 (Leung et al. 2003). Consistent with this, our single‐nucleus transcriptomic analyses reveal substantial genotype‐ and sex‐dependent transcriptional changes within hepatocytes following midlife *Ghr* disruption. Notably, hepatocytes from me 12mGHRKO mice exhibited transcriptional profiles that resembled those observed in females, indicating partial feminization of hepatic gene expression. This phenomenon has been reported in models where GH secretion patterns are experimentally altered and underscores the central role of GH in establishing and maintaining sex‐biased hepatic transcriptional networks (Zhang et al. 2012). Interestingly, our single‐nucleus transcriptomic analysis in 12mGHRKO mice revealed a reduction in hepatocyte expression of xenobiotic metabolism related genes, which appears to contrast with previous reports in germline GH‐deficient mice (Amador‐Noguez et al. 2004) that show activation of xenobiotic metabolism pathways at the bulk tissue level. Several factors may explain this apparent discrepancy. First, prior studies relied on whole tissue gene expression, which captures aggregate tissue‐level changes and may reflect contributions from multiple hepatic cell types, whereas our snRNAseq analysis specifically interrogates hepatocytes. Thus, increased xenobiotic metabolism observed in earlier models may be driven in part by non‐parenchymal cells or shifts in cellular composition, neither of which are captured when focusing exclusively on hepatocytes. Second, the timing of GHR disruption appears to be critical. Early‐life or early adult ablation (germline or 6 months) may induce compensatory upregulation of detoxification pathways, whereas disruption at midlife (12 months) may instead attenuate these pathways, potentially reflecting reduced metabolic demand, or a shift toward a more energy‐conserving state. Importantly and in line with this explanation, the reduced expression of xenobiotic metabolism genes in hepatocytes from 12mGHRKO mice may reflect a decreased requirement for basal detoxification rather than impaired capacity. Given the improved metabolic profile, enhanced insulin sensitivity, and preserved healthspan observed in these animals, attenuation of GH signaling may reduce endogenous metabolic stress and the generation of reactive or toxic byproducts. Collectively, these findings suggest that the impact of reduced GH signaling on xenobiotic metabolism is highly context‐dependent, influenced by age at intervention, cellular resolution of analysis, and sex‐specific transcriptional regulation.

The snRNAseq results also showed a reduced hepatic B‐cell proportions in both male and female 12mGHRKO mice compared to controls, indicating that midlife disruption of GH signaling also alters the hepatic immune microenvironment. This finding is notable in the context of aging, where B‐cell dynamics are increasingly recognized as contributors to immunosenescence and chronic low‐grade inflammation. Previous studies have shown that B‐cells express GHR (Gagnerault et al. 1996) and that aging is associated with functional alterations in B‐cell populations. More recent work further highlights the emergence of age‐associated B‐cell subsets that can modulate tissue inflammation and immune signaling (Bashir et al. 2026). Thus, the reduction in hepatic B‐cell abundance observed in 12mGHRKO mice may reflect reduced inflammatory burden or changes in immune cell recruitment and retention. In line with this, an increased proportion of B‐cells is found in the AT of short‐lived mice with increased GH action (Bell et al. 2023). Given the known crosstalk between endocrine signaling and immune regulation, these data suggest that attenuation of GH action in midlife may influence not only hepatocyte transcriptional programs but also tissue‐specific immune cell infiltration, potentially contributing to improved healthspan.

Metabolically, 12mGHRKO mice exhibit a “healthy obesity” phenotype, with increased adiposity but improved insulin sensitivity and reduced insulin levels (Berryman and List 2017). Importantly, the increased adiposity and reduction in lean mass observed following midlife GHR disruption were not accompanied by corresponding metabolic or functional deficits. Instead, male 12mGHRKO mice exhibited improved insulin sensitivity, preserved or improved neuromuscular performance relative to lean mass, and maintenance of vertebral trabecular bone microarchitecture. These findings indicate that the changes in body composition induced by reduced GH action should not necessarily be interpreted as physiologically detrimental and emphasize that lean and fat mass alone may not accurately reflect metabolic or musculoskeletal function in this model. Previous studies indicate that AT excess in GH action‐deficient models differs qualitatively from AT linked to metabolic disease; that is, AT in reduced GH action states is characterized by reduced fibrosis (Householder et al. 2018; List et al. 2019), senescence (Stout et al. 2014), and inflammatory signaling (Young et al. 2020) together with increased adiponectin secretion (Lubbers et al. 2013), features that collectively promote systemic insulin sensitivity (Berryman and List 2017). These favorable alterations in AT quality and secretory function provide a plausible explanation for the improved insulin‐mediated control of glycemia despite increased fat mass. In line with these results, analysis of the visceral AT of the GH‐deficient Ames mice shows increased expression of genes involved in tumor suppression, mitochondrial biogenesis, and insulin pathways (Zaczek et al. 2024). Overall, the increased fat mass observed in 12mGHRKO mice likely reflects beneficial remodeling of AT function rather than merely increased adiposity, while the preservation of physiological function despite reduced lean mass further demonstrates that these body‐composition changes do not translate directly into functional impairment. Nevertheless, deeper characterization of AT quality and muscle‐specific function in this model is important.

Consistent with the dissociation between body composition and physiological function described above, functional assessments further support the preservation of musculoskeletal function following reduced GH signaling. Male 12mGHRKO mice exhibited improved neuromuscular performance when normalized to lean mass, indicating preserved muscle function during aging despite reduced muscle mass. Consistent with this observation, multiple mouse models with reduced GH/IGF‐1 signaling, including GHRKO mice (Arum et al. 2014) and Ames dwarf mice (Ekumi et al. 2026; Johnston et al. 2025), display delayed functional decline and preservation of physiological function with aging, including sustained grip strength and motor coordination at advanced ages. However, the relationship between GH signaling and muscle function is complex and context‐dependent. For example, long‐lived Snell dwarf mice exhibit reduced muscle quality and decreased capacity for isometric torque (Rader et al. 2018). Notably, these mice also demonstrate resistance to age‐dependent maladaptation in response to resistance‐type exercise (Rader et al. 2022), suggesting that reduced GH signaling may confer resilience to specific stressors despite baseline functional limitations. Human studies examining congenital GH deficiency or GH resistance have yielded heterogeneous findings. While some cohorts have demonstrated reductions in muscle mass and physical performance (Macfarlane and Newell 2012), studies of individuals with isolated congenital GH deficiency due to a homozygous inactivating mutation in the GH releasing hormone (GHRH) receptor gene from Itabaianinha, Brazil have reported preservation of metabolic health together with several features consistent with healthy muscular function despite lifelong reductions in GH signaling (Andrade‐Guimarães et al. 2019). Remarkably, the preservation of neuromuscular function observed in 12mGHRKO mice may reflect prior exposure to physiological GH and IGF‐1 levels during development and early adulthood, which likely allows for appropriate musculoskeletal development before GH signaling is reduced, thereby mitigating some of the functional deficits observed in mice and humans with lifelong GH deficiency. Importantly, midlife suppression of GH signaling did not negatively impact skeletal aging, as vertebral trabecular microarchitecture was improved in 12mGHRKO males and preserved in females (trending to be improved). Although GH plays a well‐established role in bone remodeling, excessive GH signaling may be deleterious to skeletal and joint health as elevated GH action promotes joint degeneration and chondrocyte metabolic dysfunction (Zhu et al. 2023), whereas disruption of GH receptor signaling reduces osteoarthritis severity and chondrocyte hypertrophy (Liu et al. 2024). Consistent with these findings, GH overexpression accelerates intervertebral disc degeneration, while GH antagonism attenuates these degenerative changes (Sukul et al. 2025). Collectively, these findings suggest that targeting GH signaling later in life preserves neuromuscular and skeletal structure while minimizing the developmental and physiological trade‐offs associated with lifelong GH suppression.

An important finding of the present study is the clear sexual dimorphism in the response to midlife GHR disruption. Although female mice exhibited a significant extension of lifespan, improvements in the functional healthspan outcomes evaluated in this study were largely absent. Female mice generally exhibit slower aging trajectories, lower age‐related mortality, and greater longevity than males, potentially limiting the ability to detect additional improvements in physiological function. These findings suggest that the mechanisms regulating lifespan and healthspan may differ between the sexes and are consistent with previous studies demonstrating that both sex chromosomes and gonadal hormones independently influence aging trajectories. For example, studies using the Four Core Genotypes mouse model have shown that sex chromosome complement contributes to longevity, with XX mice exhibiting greater survival than XY mice independent of gonadal sex (Davis et al. 2019). Although subsequent characterization of this model identified structural alterations associated with the modified Y chromosome (Panten et al. 2024), these studies nevertheless support the concept that intrinsic sex‐specific genetic factors contribute to longevity. In addition to differences in sex chromosome complement and gonadal hormones, males and females exhibit distinct patterns of GH secretion (Jansson et al. 1985) that result in sexually dimorphic downstream GH signaling and gene expression. These differences likely contribute to the distinct physiological responses to reduced GH action observed in the present study and are discussed in greater detail below. Importantly, the absence of measurable improvements in the specific healthspan outcomes evaluated here should not be interpreted as evidence that midlife GHR disruption fails to improve health in female mice. Rather, it suggests that the longevity benefit in females may be mediated through physiological mechanisms not captured by the endpoints examined in the present study. Future studies evaluating additional domains of healthspan, including immune function, cognitive function, resilience to physiological stress, and immune aging, will be important to determine whether female mice experience benefits in aspects of healthspan not assessed here. Altogether, these findings highlight the importance of considering sex as a biological variable when evaluating interventions targeting GH signaling and aging.

In summary, our findings demonstrate that suppression of GH signaling beginning in middle age is sufficient to extend lifespan in both sexes while preserving or improving selected aspects of metabolic and functional health, particularly in males. These results further demonstrate that developmental GH suppression is not required to obtain longevity benefits, indicating that modulation of GH signaling during adulthood remains sufficient to influence aging trajectories. Importantly, the sexually dimorphic responses observed in the present study suggest that the effects of reduced GH action on healthspan are likely tissue‐ and sex‐specific and emphasize the importance of considering sex as a biological variable when developing future gerotherapeutic interventions. Our data further suggest that reprogramming of hepatic metabolic and endocrine gene networks may represent a key mechanism through which midlife GH suppression influences systemic metabolism and longevity. Collectively, these findings reinforce the central role of the GH/IGF‐1 axis in aging biology and support the concept that pharmacological inhibition of GH signaling during adulthood may represent a feasible strategy for promoting healthy aging. From a translational perspective, these findings are particularly compelling because pharmacological inhibition of GH signaling is already clinically feasible. GHR antagonism is currently FDA‐approved for the treatment of acromegaly and effectively suppresses GH signaling in humans. In addition, next‐generation GHR antagonists and alternative formulations designed to improve clinical practicality, including long‐acting injectable agents and small‐molecule inhibitors, are currently under development (Basu et al. 2023), further expanding the translational potential of this therapeutic strategy. Given the growing body of evidence linking reduced GH/IGF‐1 signaling to improved metabolic health and extended longevity across multiple species, pharmacological modulation of this pathway represents a promising strategy for promoting healthy aging. Future studies should define the optimal timing, dosage, and duration of GH‐targeted interventions, determine the latest therapeutic window at which reduced GH action remains effective, and evaluate their long‐term effects on resilience, sex‐specific healthspan outcomes, and age‐related disease risk.

## 4 Experimental Procedures

### 4.1 Generation and Maintenance of 12mGHRKO Mice

Mice carrying LoxP sites flanking exon 4 of the Ghr gene on a C57BL/6J background were generated by the Knockout Mouse Project (KOMP) as previously described (Junnila et al. 2016). These mice were crossed with tamoxifen‐inducible Cre recombinase mice under the control of the ubiquitous ROSA26 promoter (B6.129‐Gt(ROSA)26Sortm1(cre/ERT2)Tyj/J; Jackson Laboratory). Mice were bred to homozygosity for the floxed *Ghr* allele and Cre recombinase. To induce *Ghr* deletion, 12‐month‐old mice received intraperitoneal injections of tamoxifen dissolved in peanut oil (12mGHRKO) or vehicle (control). Tamoxifen was administered at a total dose of 0.32 mg/g body weight over five consecutive days as previously described (Junnila et al. 2016).

Three cohorts of male and female mice were used. Two experimental cohorts were used for phenotypic and molecular characterization and were sacrificed at 19 or 23 months of age (*n* = 9–12 per group). A third cohort was used for longevity studies and animals were followed until natural death (*n* = 30–35 per group). No animals died prior to induction at 12 months of age. Therefore, the experimental cohorts were not subject to survival‐based selection before initiation of GHR deletion. Mice were housed at 22°C under a 14‐h light/10‐h dark cycle with ad libitum access to water and standard chow (ProLab RMH 3000). All procedures were approved by the Ohio University Institutional Animal Care and Use.

### 4.2 Serum Collection and Tissue Dissection

Blood was collected from the orbital sinus and blood was left to clot for 30 min at room temperature. Clotted blood was then centrifuged at 1000–2000×*g* for 10 min to separate the clot from the liquid supernatant. For tissue collection, mice were fasted overnight prior to euthanasia. Animals were euthanized by CO_2_ inhalation followed by cervical dislocation. Organs were dissected, weighed, snap frozen in liquid nitrogen, and stored at −80°C.

### 4.3 Validation of GHR Disruption

*Ghr* deletion was confirmed using RT‐qPCR to assess *Ghr* and *Igf‐1* expression in liver, subcutaneous AT, perigonadal AT, kidney, quadriceps muscle, and heart. Circulating GH and IGF‐1 concentrations were measured by ELISA (Alpco Mouse/rat GH or IGF‐1 cat. 22‐GHOMS‐E01 and 22‐IG1MS‐E01).

### 4.4 RT‐qPCR

RNA was isolated from frozen tissues using the GeneJET RNA Purification Kit (Thermo Scientific). Tissues were homogenized using a Precellys 24‐Dual homogenizer. RNA concentration and purity were assessed using a NanoDrop 2000c spectrophotometer and only samples with 260/280 and 260/230 ratios ≥ 1.8 were used.

cDNA was synthesized using the Maxima First Strand cDNA Synthesis Kit and quantitative PCR was performed using Power SYBR (Thermo Scientific) on a Bio‐Rad iCycler. Gene expression was normalized to two housekeeping genes using qBasePlus software (Biogazelle).

### 4.5 Hormone Measurements

Serum GH and IGF‐1 levels were measured using mouse/rat GH (22‐GHOMS‐E01) and mouse/rat IGF‐1 (22‐IG1MS‐E01) ELISA kits (ALPCO) according to manufacturer instructions. Insulin, leptin, resistin, interleukin‐6 (IL‐6), and monocyte chemoattractant protein‐1 (MCP‐1) were determined in serum using MILLIPLEX MAP Mouse Metabolic Hormone Magnetic Bead Panel kit (MMHMAG‐44 K) on a Milliplex 200 Analyzer (Millipore), following manufacturer's instructions.

### 4.6 Body Composition and Length

Body composition was measured monthly using a Bruker Minispec NMR analyzer starting 1 day before tamoxifen administration and continuing until sacrifice. Fat mass, lean mass, and fluid content were determined. Body length was measured from nose to anus.

### 4.7 Glucose Metabolism

Glucose tolerance tests (GTT) and insulin tolerance tests (ITT) were performed at 19 and 23 months of age. For GTT, mice were fasted overnight and injected intraperitoneally with 10% glucose (0.01 mL/g body weight). For ITT, mice were fasted for 6 h and injected with recombinant human insulin (Novolin‐R, Novo Nordisk). Blood glucose levels were measured using OneTouch Ultra glucometers at baseline and at 15, 30, 45, 60, and 90 min after injection.

### 4.8 Healthspan Assessments

#### 4.8.1 Rotarod Test

Motor coordination and balance were assessed using an accelerating rotarod apparatus (Ugo Basile). Mice were acclimated to the apparatus for two consecutive days prior to testing, during which they were placed on the rotating rod at a constant low speed (4 rpm) for up to 2 min per trial. For testing, mice were placed on a rotating rod accelerating from 4 to 40 rpm over 5 min. Latency to fall was recorded. Each mouse performed three trials per session, and the average latency was used for analysis.

#### 4.8.2 Grip Strength Test

Forelimb and hindlimb grip strength were measured using a grip strength meter (Columbus Instruments). Mice grasped a metal grid and were gently pulled backwards until release. Five measurements were obtained per mouse and averaged. Grip strength values were normalized to lean mass measured by NMR.

#### 4.8.3 Frailty Index

Frailty was assessed using a validated mouse frailty index based on the deficit accumulation model (Parks et al. 2012). Animals were scored across multiple health parameters including coat condition, body condition, gait disorders, hearing loss, tremor, and ocular abnormalities. Each parameter was scored as 0 (absent), 0.5 (mild), or 1 (severe). Frailty scores were calculated as the ratio of deficits observed relative to the total number of parameters assessed.

#### 4.8.4 Bone Micro–Computed Tomography (μCT)

Lumbar vertebrae (L3) were scanned ex vivo using a SCANCO μCT35 system (SCANCO Medical AG, Bassersdorf, Switzerland) following standard protocols for rodent bone analysis. Lumbar vertebral bones (L5) were dissected after euthanasia, stored at −80°C, fixed in 4% paraformaldehyde before subsequently imaged at a voxel size of 7–10 μm, appropriate for evaluating trabecular bone architecture. Scans were acquired using manufacturer‐recommended settings (55 kVp, 114 μA, 900 ms integration time), with voltage and current maintained within the 45–70 kVp operating range to ensure consistent image quality across specimens. Three‐dimensional reconstructions were generated using SCANCO Evaluation software, and regions of interest (ROIs) encompassing whole L3 were quantitatively analyzed for bone morphometry. Quantitative bone parameters, including bone volume fraction (BV/TV), trabecular thickness (Tb.Th), trabecular number (Tb.N), trabecular separation (Tb.Sp), bone mineral density (BMD), and Vertebrate Height (Vert. Height) were obtained using manufacturer‐recommended algorithms.

### 4.9 Single‐Nucleus RNA Sequencing (snRNA‐Seq)

Frozen liver tissue from 19‐month‐old mice was used for snRNA‐seq (*n* = 4/genotype). Tissue was homogenized and nuclei were purified through filtration and centrifugation using the Sigma EZ Nuclei isolation Kit #NUC‐101. Nuclei were counted and sent to USC‐Buck Nathan Shock Center for processing using the 10× Genomics Chromium platform for library preparation using the Chromium Single Cell 3′ Gene Expression kit.

Libraries were sequenced on an Illumina platform. Raw sequencing data were processed using Cell Ranger (10× Genomics) for demultiplexing, alignment to the mm10 mouse genome, and generation of gene‐cell count matrices.

#### 4.9.1 snRNA‐Seq Bioinformatic Analysis

Downstream analysis was performed using the Seurat (v4) R package. Cells were filtered to have greater than 200 and fewer than 2500 unique molecule identifiers and less than 5% mitochondrial unique molecule identifiers. Genes appearing in fewer than 3 cells were filtered out of the dataset. The gene counts were normalized using LogNormalize and linear principal component analysis (PCA) was performed to determine dimensionality. Clusters were categorized using a dimensionality of 17 and a resolution of 0.35.

Cell populations were visualized using Uniform Manifold Approximation and Projection (UMAP). The predominant cell type in each cluster was determined using SingleR 2.12.2 (Aran et al. 2019). Expression data from Pietilä et al. (Pietilä et al. 2025) were used as the reference annotation, and results were pruned with nmads = 0.5 to remove uncertain labels. Differential gene expression analyses were performed using Seurat's FindMarkers function. Sequencing quality metrics indicated recovery of approximately 5000–10,000 nuclei per sample, with 30,000–50,000 reads per nucleus and ~1500–3000 genes detected per nucleus after quality filtering.

### 4.10 Histopathology

At the time of death, mice were fixed in 10% formalin and submitted to the University of Texas at San Antonio Pathology Core for histopathological analysis (Ikeno et al. 2005). Tissues were paraffin‐embedded, sectioned, and stained with hematoxylin and eosin (H&E). Slides were evaluated independently by two pathologists blinded to experimental groups. Lesions were classified according to established criteria for aging mice, and both neoplastic and non‐neoplastic findings were recorded for each animal. Tumor burden was defined as the total number of distinct tumor types per mouse, while disease burden reflected the total number of histopathological lesions identified. Severity of neoplastic and renal lesions was graded using a standardized scoring system. Mice with high‐grade neoplastic lesions (Grade 3–4) were classified as having died from neoplastic disease. Concordance between pathologists exceeded 90%; in cases of disagreement or when lesions were not considered sufficient to explain mortality, cause of death was classified as undetermined (Ikeno et al. 2009).

### 4.11 Statistical Analysis

Data are presented as mean ± SE. Statistical analyses were performed using GraphPad Prism. Student's two‐tailed *t*‐tests were used to compare genotypes within sex. Two‐way ANOVA was used to evaluate genotype and sex effects. Repeated‐measures ANOVA was used for longitudinal measurements. Survival curves were analyzed using log‐rank and Gehan‐Breslow‐Wilcoxon tests. Median and maximal lifespan were evaluated using Fisher's exact test. Survival analysis was performed using OASIS 2. To complement the Kaplan–Meier percentile analyses, maximal lifespan was additionally evaluated using the method described by Wang et al. (2004). For each sex, survival data from control and 12mGHRKO mice were pooled to determine the age corresponding to the 90th percentile survival, which defined the longest‐lived 10% of animals. The proportion of mice surviving to or beyond this cutoff was compared between genotypes using Fisher's exact test. Statistical significance was defined as *p* < 0.05.

## Author Contributions

Study conception/design: Silvana Duran‐Ortiz, John J. Kopchick. Acquisition of data: Silvana Duran‐Ortiz, Yuji Ikeno, Shouan Zhu, Patrick M. O'Connor, Bérénice A. Benayoun, Todd McHugh, Fabian Benencia, Minhoo Kim. Analysis and interpretation of data: Silvana Duran‐Ortiz, Edward O. List, Yuji Ikeno, Reetobrata Basu, Emmanuel A. Gotte, Darlene E. Berryman, Jonathan A. Young. Manuscript preparation: Silvana Duran‐Ortiz, John J. Kopchick, Emmanuel A. Gotte.

## Funding

This work was supported by the State of Ohio's Eminent Scholar Program that includes a gift from Milton and Lawrence Goll, by NIH grant R01AG059779 to J.J.K. and R01AG070034 to Y.I., The American Federation of Aging Research (Glenn/AFAR) postdoctoral fellowship to S.D.O., The pilot award from USC‐Buck Nathan Shock Center (NIA P30 AG068345) to S.D.O., the pilot award from San Antonio Nathan Shock Center Pathology core (NIA P30 AG068345) to S.D.O. and by the AMVETS, and by the Diabetes Institute at Ohio University.

## Ethics Statement

Animal protocol was approved by the Institutional Animal Care and Use Committees of Ohio University.

## Conflicts of Interest

The authors declare no conflicts of interest.

## Supporting information


**Table S1:** Median and percentile survival estimates for experimental cohorts. Survival summary statistics for each experimental group derived from Kaplan–Meier analysis. Percentile columns indicate the estimated time (in days) at which 25%, 50%, 75%, 90% and 100% mortality was reached for each cohort. The final column provides the 95% confidence interval for median survival.
**Table S2:** Pairwise survival comparisons at mortality percentiles among control and 12mGHRKO cohorts pairwise comparisons of survival distributions among female control, female 12mGHRKO, male control, and male 12mGHRKO mice at defined mortality percentiles (25%, 50%, 75%, and 90%). This analysis was used to assess whether the effects of midlife *Ghr* disruption differed by sex and across early‐, mid‐, and late‐life survival intervals. Statistical significance was calculated using Fischer exact test, *p* < 0.05.
**Table S3:** Incidence of fatal and total pathologic lesions in control and 12mGHRKO mice summary of end‐of‐life pathology outcomes in male and female control and 12mGHRKO mice. Fatal lesions indicate the primary pathology determined to be the cause of death, whereas total lesions include all observed cases regardless of whether the lesion was considered fatal. Comparisons within each sex were analyzed using Fisher's exact test, (*p* < 0.05) are highlighted. Control (*n* = 25) and 12mGHRKO (*n* = 18); females, control (*n* = 20) and 12mGHRKO (*n* = 27).
**Table S4:** Distribution of major liver cell populations and hepatocyte subclusters by sex and genotype in 12mGHRKO and control Mice. Percent composition of nuclei assigned to major hepatic cell populations and hepatocyte subclusters identified by single‐nucleus RNA sequencing (snRNA‐seq) of livers from female control (F Control), female 12mGHRKO (F 12mGHRKO), male control (M Control), and male 12mGHRKO (M 12mGHRKO) mice. Hepatocyte subclusters were generated based on similarity of gene expression profiles.
**Table S6:** Maximal lifespan analysis using the Wang et al. (2004) method. Maximal lifespan was evaluated using the method of Wang et al. (2004). For each sex, survival data from control and 12mGHRKO mice were pooled to determine the age corresponding to the 90th percentile survival (longest‐lived 10% of animals). The proportion of animals surviving beyond this cutoff was compared between genotypes using Fisher's exact test.
**Figure S1:** *Ghr* and *Igf‐1* gene expression across multiple tissues inmale and female 12mGHRKO and control mice. (a, b) *Ghr* gene expression at 19 (a) and 23 (b) months of age (*n* = 9–12/group). (c, d) *Igf‐1* gene expression at 19 (c) and 23 (d) months of age (*n* = 9/group). *Ghr* and *Igf‐1* mRNA levels were determined by RT‐qPCR. All values are mean ± SE. Student two‐tailed T tests were used to assess differences between 2 groups (female or male 12mGHRKO mice vs. controls). *p ≤ 0.05. Subq, subcutaneous; Quad, quadriceps.
**Figure S2:** Longitudinal body weight and body composition following GHR disruption in middle‐aged male and female mice. (a and e) Body weight over time in male (a) and female (e) 12mGHRKO and control mice. (b–h) Fat (b, f), lean (c, g), and fluid mass (d, h) over time in male and female 12mGHRKO and control mice (*n* = 9–12/group). All values are mean ± SE. Repeated Measures ANOVA was used to assess differences over time. *p ≤ 0.05.
**Figure S3:** Serum inflammatory markers in male and female 12mFGRKO and control mice at 19 months of age. Circulating levels of (a) IL‐6, (b) MCP‐1, (c) TNF‐a, (d) leptin and (e) Resistin in male and female 12mGHRKO and control mice (*n* = 9/group). Student two‐tailed T test was used to assess significant differences between experimental and control mice of the same sex. All values are mean ± SE. *p ≤ 0.05.
**Figure S4:** Total and normalized tissue weights of male and female 12mGHRKO and control mice at 19‐ and 23‐months of age. (a) Relative organ weight at the time of dissection (*n* = 8–12/group). (b) Total organ weight at the time (*n* = 7–12/group). Organ weights were normalized to body weight. All values are mean ± SE. * *p* ≤ 0.05. Sc, subcutaneous; Peri, perigonadal; Bat. Brown adipose tissue; Gas, gastrocnemius; Sol, soleus; Quad, quadriceps.
**Figure S5:** Improved insulin sensitivity and normal glucose tolerance at 19 months of age in 12mGHRKO males. (a, b) Glucose tolerance tests (GTTs) in male and female 12mGHRKO mice versus controls. (c, d) Insulin tolerance tests (ITTs) in male and female 12mGHRKO mice versus controls. Student *T* test for difference between individual time points for each sex. Black bars represent male and red bars female controls, whereas grey and white bars represent male and female 6mGHRKO mice, respectively. All values are mean ± SE. Repeated Measures ANOVA was used to assess differences over time. *p ≤ 0.05.
**Figure S6:** Health span assessment of male and female 12mGHRKO and control mice over time. (a–d) Rotarod performance in male (a) and female (d) mice over time. (b–e) Forelimb grip strength in male (b) and female (e) mice over time. (c–f) Frailty index scores in male (c) and female (f) mice assessed longitudinally with age. Black symbols represent control mice, whereas grey symbols represent 12mGHRKO mice. All values are mean ± SE. Repeated measures two‐way ANOVA was used to evaluate differences between groups over time. *p ≤ 0.05.

**Table S5:** Pathway analysis of 12mGHRKO male and female hepatocyte clusters.

## Data Availability

Datasets generated and analyzed are available upon request. Our studies do not include the use of custom code or mathematical algorithms.
