# Supplementary material for: Midlife Growth Hormone Receptor Ablation Extends Healthy Lifespan and Induces Sex‐Specific Hepatic Transcriptional Changes at Single‐Cell Resolution
Source: Aging Cell. 2026 Sep 5;25(9):e70695. doi: 10.1111/acel.70695 (PMC13545643; doi:10.1111/acel.70695)
Supplement: Supplementary file 1 [file ACEL-25-e70695-s001.docx]

**Supplementary Table 1.**
**Median and Percentile Survival Estimates for Experimental Cohorts**.
Survival summary statistics for each experimental group derived from Kaplan–Meier analysis. Percentile columns indicate the estimated time (in days) at which 25%, 50%, 75%, 90%, and 100% mortality was reached for each cohort. The final column provides the 95% confidence interval for median survival.

**Supplementary Table 2.**
**Pairwise Survival Comparisons at Mortality Percentiles Among Control and 12mGHRKO Cohorts**
Pairwise comparisons of survival distributions among female control, female 12mGHRKO, male control, and male 12mGHRKO mice at defined mortality percentiles (25%, 50%, 75%, and 90%).. This analysis was used to assess whether the effects of midlife *Ghr* disruption differed by sex and across early-, mid-, and late-life survival intervals. Statistical significance was calculated using Fischer exact test, *P* < 0.05.

**Supplementary Table 3.**
**Incidence of Fatal and Total Pathologic Lesions in Control and 12mGHRKO Mice**
Summary of end-of-life pathology outcomes in male and female control and 12mGHRKO mice. Fatal lesions indicate the primary pathology determined to be the cause of death, whereas total lesions include all observed cases regardless of whether the lesion was considered fatal. Comparisons within each sex were analyzed using Fisher’s exact test, (*P* < 0.05) are highlighted. Control (*n* = 25) and 12mGHRKO (*n* = 18); females, control (*n* = 20) and 12mGHRKO (*n* = 27).

**Supplemental Fig. 1**. ***Ghr* and *Igf-1* Gene Expression Across Multiple Tissues inmale and female 12mGHRKO and control mice. (**a-b) *Ghr* gene expression at 19 (a) and 23 (b) months of age (n = 9-12/group). (c-d) *Igf-1* gene expression at 19 (c) and 23 (d) months of age (n = 9/group). *Ghr* and *Igf‐1* mRNA levels were determined by RT-qPCR. All values are mean ± SE. Student two‐tailed T tests were used to assess differences between 2 groups (female or male 12mGHRKO mice vs. controls). **p* ≤ 0.05. Subq, subcutaneous; Quad, quadriceps

**Supplemental Fig. 2**. **Longitudinal body weight and body composition following GHR disruption in middle-aged male and female mice**. (a and e) Body weight over time in male (a) and female (e) 12mGHRKO and control mice. (b -h) Fat (b, f), lean (c, g), and fluid mass (d, h) over time in male and female 12mGHRKO and control mice (n=9-12/group). All values are mean ± SE. Repeated Measures ANOVA was used to assess differences over time. **p* ≤ 0.05.

**Supplemental Fig. 3. Serum inflammatory markers in male and female 12mFGRKO and control mice at 19 months of age.** Circulating levels of a) IL-6, b) MCP-1, c) TNF-a, d) leptin and e) Resistin in male and female 12mGHRKO and control mice (n = 9/group). Student two‐tailed T test was used to assess significant differences between experimental and control mice of the same sex. All values are mean ± SE. **p* ≤ 0.05

**Supplementary Fig 4. Total and normalized tissue weights of male and female 12mGHRKO and control mice at 19- and 23-months of age**. a) Relative organ weight at the time of dissection (n=8-12/group). b) Total organ weight at the time (n=7-12/group). Organ weights were normalized to body weight. All values are mean ± SE. * P ≤ 0.05. Sc, subcutaneous; Peri, perigonadal; Bat. Brown adipose tissue; Gas, gastrocnemius; Sol, soleus; Quad, quadriceps.

**Supplemental Figure 5. Improved insulin sensitivity and normal glucose tolerance at 19 months of age in 12mGHRKO males .** a-b) Glucose tolerance tests (GTTs) in male and female 12mGHRKO mice vs. controls. c-d) Insulin tolerance tests (ITTs) in male and female 12mGHRKO mice vs. controls. Student T test for difference between individual time points for each sex. Black bars represent male and red bars female controls, whereas grey and white bars represent male and female 6mGHRKO mice, respectively All values are mean ± SE. Repeated Measures ANOVA was used to assess differences over time. **p* ≤ 0.05.

**Supplemental Figure 6. Health span assessment of male and female 12mGHRKO and control mice over time.** a-d) Rotarod performance in male (a) and female (d) mice over time. b-e) Forelimb grip strength in male (b) and female (e) mice over time. c-f) Frailty index scores in male (c) and female (f) mice assessed longitudinally with age. Black symbols represent control mice, whereas grey symbols represent 12mGHRKO mice. All values are mean ± SE. Repeated measures two-way ANOVA was used to evaluate differences between groups over time. *P ≤ 0.05.

**Supplementary Table 4.**
**Distribution of major liver cell populations and hepatocyte subclusters by sex and genotype in 12mGHRKO and control Mice.**
Percent composition of nuclei assigned to major hepatic cell populations and hepatocyte subclusters identified by single-nucleus RNA sequencing (snRNA-seq) of livers from female control (F Control), female 12mGHRKO (F 12mGHRKO), male control (M Control), and male 12mGHRKO (M 12mGHRKO) mice. Hepatocyte subclusters were generated based on similarity of gene expression profiles.

**Supplementary Table 6.**

**Maximal Lifespan Analysis Using the Wang et al. (2004) Method.**

Maximal lifespan was evaluated using the method of Wang et al. (2004). For each sex, survival data from control and 12mGHRKO mice were pooled to determine the age corresponding to the 90th percentile survival (longest-lived 10% of animals). The proportion of animals surviving beyond this cutoff was compared between genotypes using Fisher's exact test.
